# Does previous gastrectomy history affect the surgical outcomes of laparoscopic cholecystectomy?

**DOI:** 10.1186/s12893-023-02237-7

**Published:** 2023-10-23

**Authors:** Xin-Peng Shu, Ze-Lin Wen, Qing-Shu Li

**Affiliations:** 1 Department of Gastrointestinal Surgery, The First Affiliated Hospital of Chongqing Medical University, Chongqing, 400016 China; 2 Department of Gastrointestinal Surgery, Chongqing Medical University, Yongchuan Hospital, Chongqing, 402160 China; 3 Department of Pathology, College of Basic Medicine, Chongqing Medical University, Chongqing, China; 4 Molecular Medicine Diagnostic and Testing Center, Chongqing Medical University, Chongqing, China; 5 Department of Pathology, the First Affiliated Hospital of Chongqing Medical University, Chongqing, China

**Keywords:** Laparoscopic cholecystectomy, Gastrectomy, Outcomes

## Abstract

**Purpose:**

This current study aimed to explore whether gastrectomy history influenced surgical outcomes while undergoing laparoscopic cholecystectomy (LC).

**Methods:**

The PubMed, Embase, and Cochrane Library databases were searched for eligible studies from inception to April 29, 2023. The Newcastle–Ottawa Scale (NOS) was adopted to assess the quality of included studies. The mean differences (MDs) and 95% confidence intervals (CIs) were calculated for continuous variables, and the odds ratios (ORs) and 95% CIs were calculated for dichotomous variables. RevMan 5.4 was used for data analysis.

**Results:**

Seven studies enrolling 8193 patients were eligible for the final pooling up analysis (380 patients in the previous gastrectomy group and 7813 patients in the non-gastrectomy group). The patients in the gastrectomy group were older (MD = 11.11, 95%CI = 7.80–14.41, *P* < 0.01) and had a higher portion of males (OR = 3.74, 95%CI = 2.92–4.79, *P* < 0.01) than patients in the non-gastrectomy group patients. Moreover, the gastrectomy group had longer LC operation time (MD = 34.17, 95%CI = 25.20–43.14, *P* < 0.01), a higher conversion rate (OR = 6.74, 95%CI = 2.17–20.26, *P* = 0.01), more intraoperative blood loss (OR = 1.96, 95%CI = 0.59–3.32, *P* < 0.01) and longer postoperative hospital stays (MD = 1.07, 95%CI = 0.38–1.76, *P* < 0.01) than the non-gastrectomy group.

**Conclusion:**

Patients with a previous gastrectomy history had longer operation time, a higher conversion rate, more intraoperative blood loss, and longer postoperative hospital stays than patients without while undergoing LC. Surgeons should pay more attention to these patients and make prudent decisions to avoid worse surgical outcomes as much as possible.

## Introduction

Laparoscopic cholecystectomy (LC) was a standard treatment of gallbladder disease including symptomatic cholecystolithiasis, especially the acute cholecystitis [1]. Compared with open cholecystectomy (OC), studies showed that LC had a mild incision, shorter postoperative hospital stays, fewer postoperative complications, and more enhanced recovery [2–4]. However, previous upper abdominal surgery history might increase the conversion rate from LC to OC [5], which was considered a relative contraindication in the past decades [6–8].

Gastrectomy was the main treatment for gastric cancer, including distal, total, proximal, and partial gastrectomy [9–11]. Some studies have demonstrated that patients with a previous gastrectomy history had an increaseed incidence of cholecystolithiasis [12, 13]. However, after the primary gastrectomy surgery, the peritoneal adhesion might increase the difficulties for the second LC surgery [14, 15].

There existed an argument about whether previous gastrectomy history affected the surgical events while patients undergoing LC. Some studies revealed that previous gastrectomy increased the conversion rate while undergoing LC [16–19]. However, others reported opposite [20, 21]. Therefore, the purpose of this current study was to evaluate the effect of a previous gastrectomy history on the surgical outcomes in patients who underwent LC.

## Materials and methods

This study was conducted according to the Preferred Reporting Items for Systematic Reviews and Meta Analyses (PRISMA) statement [22]. The registration ID of this study on PROSPERO was CRD42023465540, and the link washttps://www.crd.york.ac.uk/prospero/display_record.php?ID=CRD42023465540.

### Search strategy

The PubMed, Embase, and Cochrane Library were searched for eligible studies from inception to April 29, 2023. The search strategy included “gastrectomy” and “laparoscopic cholecystectomy”. In terms of gastrectomy, we used “gastrectomy” OR “stomach resection” OR “gastric resection” OR “stomach surgery” OR “gastric surgery” to expand the search scope. As for “laparoscopic cholecystectomy”, we used “laparoscopic cholecystectomy” OR “laparoscopy cholecystectomy”. The two main items were combined with “AND”, and the search scope was limited to “the Title and Abstract”. The search language was restricted to English, and two authors conducted this search independently.

### Inclusion and exclusion criteria

The inclusion criteria were as follows: (1), Studies that identified patients who underwent LC; (2), Studies that divided the LC patients into the gastrectomy group and the non-gastrectomy group; (3), Studies which compared the surgical outcomes between the two groups. The exclusion criteria were as follows: (1), Incomplete data about the surgical outcomes; (2), Case reports, case series, letters to the editor, comments, conferences, and reviews.

### Study selection

According to the Inclusion and exclusion criteria, the selection procedure was performed by two authors, respectively. First, the duplicated studies among the three databases would be excluded. Second, the titles and abstracts were screened for eligible studies. Then, the full texts were assessed for final analysis. Disagreements were solved by the third author.

### Data collection

The baseline information, including the identified studies and the identified patients, were extracted by two authors, respectively. The baseline characteristics of the included studies were as follows: first author, year of publication, country of study, study date, sample size, and study type. The patients’ information included age, sex, and gallbladder status et al. The surgical outcomes including the operation time, intraoperative blood loss, conversion rate, and postoperative hospital stays were also collected.

### Quality assessment

The quality assessment of the included studies was conformed to the Newcastle–Ottawa Scale (NOS) [23]. A study with nine points represented high-quality. A study with seven to eight points was considered middle-quality, and a study with less than seven points was defined as low-quality.

### Statistical analysis

Continuous variables including age, operation time, and postoperative hospital stays were calculated by the mean differences (MDs) and 95% confidence intervals (CIs). The odds ratios (ORs) and 95% CIs were calculated for dichotomous variables. The heterogeneity of identified research was evaluated by the I^2^ value. The I^2^ > 50% indicated high heterogeneity, and the random effects model was adopted, and *P* < 0.1 was considered statistical difference. The fix effects model was used when the I^2^ < 50%, which represented low heterogeneity, and *P* < 0.05 meant statistically significant. The funnel plot was used to assess the publication bias. The RevMan 5.4 (The Cochrane Collaboration, London, United Kingdom) was performed for this data analysis.

## Results

### Study selection

**Fig. 1:**
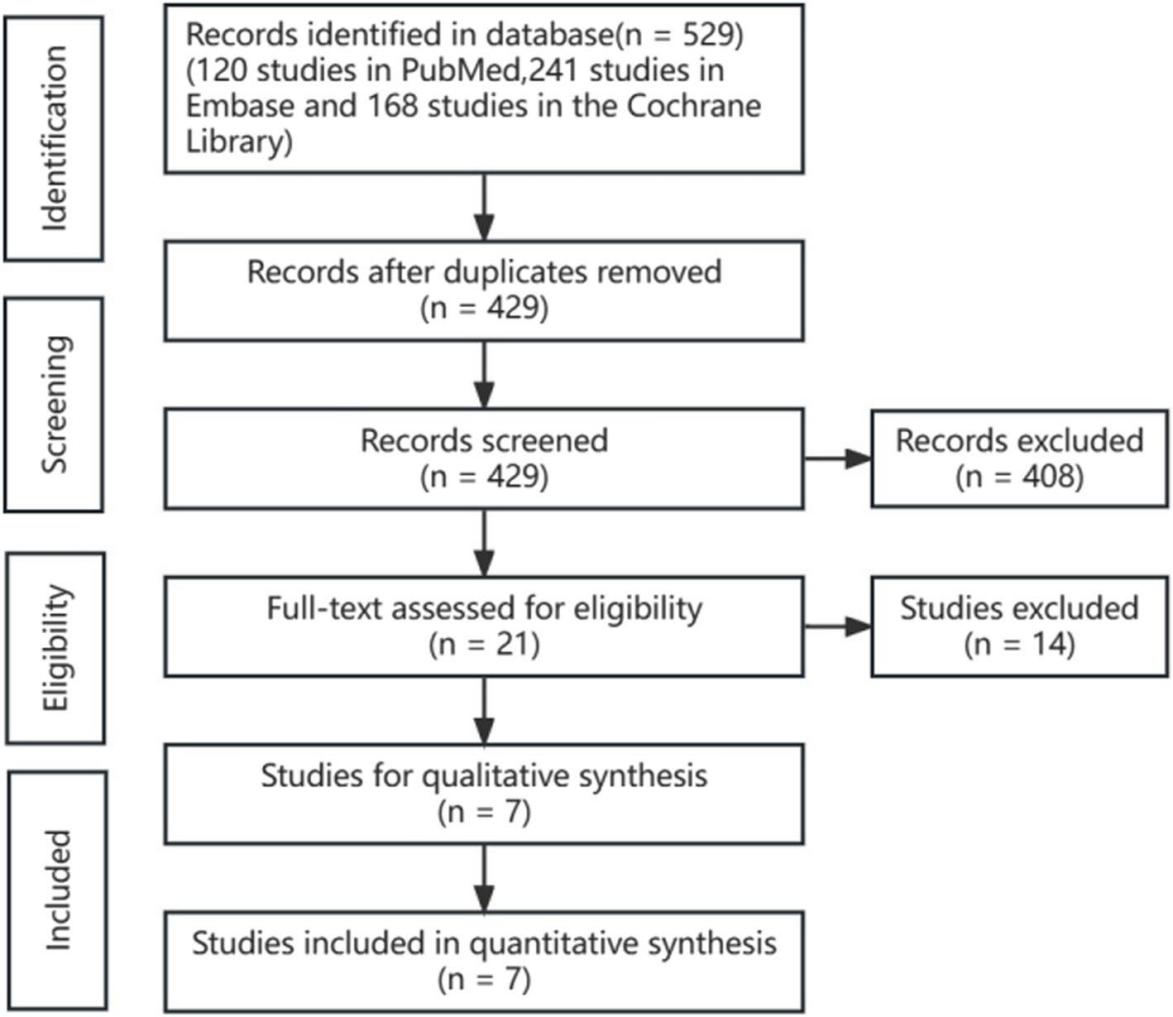
Flow chart of study selection

Totally, 529 studies were identified according to the search strategy (120 studies in PubMed, 241 studies in Embase and 168 studies in the Cochrane Library). 100 duplicated studies were excluded initially. Then, the titles and abstracts of the 429 studies were screened, and 21 studies were left for full-text screening. After the full-text screening, seven studies [16–21, 24] were identified for final analysis. No more eligible studies were found by reviewing the reference of the included seven studies. The flow chart of study selection was shown in Fig. 1.

### Baseline information of included studies

A total of seven studies including 8193 patients were identified for this study. The publish dates of these studies were from 2008 to 2021. The regions of these studies included Japan (three studies), China (two studies), Korea (one study) and Canada (one study). Six of them were retrospective studies and one was designed prospectively. More baseline information and the NOS score were shown in Table 1.

**Table 1:** Baseline characteristics of included studies

Author	Year	Country	Study date	Patients	Study type	Gastrectomy/Non-gastrectomy	NOS
Lee DH	2021	Korea	2012–2019	1258	prospectively	77/1141	8
Sasaki A	2008	Japan	1992–2007	1104	retrospectively	51/1053	7
Shannon A	2009	Canada	1990–2005	1137	retrospectively	14/1123	8
Wang ML	2013	China	2003–2010	60	retrospectively	30/30	7
Zhang MJ	2016	China	2010–2015	1022	retrospectively	127/895	7
Harino T	2021	Japan	2008–2019	2235	retrospectively	39/2196	8
Yamamoto H	2013	Japan	1991–2007	2004	retrospectively	42/1375	7

*Abbreviations*: *NOS* Newcastle–Ottawa Scales

### Summary of characteristics between gastrectomy group and non-gastrectomy group

Age, sex, gallbladder status, intraoperative biliary injury, and postoperative complications were showed in Table 2. The results showed that patients in the gastrectomy group were older (MD = 11.11, 95%CI = 7.80–14.41, P < 0.01) and had a higher portion of males (OR = 3.74, 95%CI = 2.92–4.79, *P* < 0.01) than the non-gastrectomy group. Moreover, the gastrectomy group had a higher portion of common bile duct (CBD) stone (OR = 3.67, 95%CI = 2.58–5.20, *P* < 0.01), bile leakage (OR = 19.00, 95%CI = 5.44–66.41, *P* < 0.01), and wound infection (OR = 11.23, 95%CI = 3.75–33.67, *P* < 0.01) than the non-gastrectomy group.

**Table 2:** Summary meta-analysis of comparison between gastrectomy group and non-gastrectomy group

Characteristics	Studies	Participants (Gastrectomy/Non-gastrectomy)	Odds Ratio/Mean Difference (95% CI)	Heterogeneity
Age	7	380/7813	11.11 [7.80, 14.41]; *P* < 0.01	I^2^ = 90%; *P* < 0.01
Sex (male)	6	350/7783	3.74 [2.92, 4.79]; *P* < 0.01	I^2^ = 29%; *P* = 0.22
Gallbladder status				
Acute or chronic cholecystitis	4	95/1136	1.20 [0.83, 1.75]; *P* = 0.33	I^2^ = 9%; *P* = 0.35
Symptomatic cholelithiasis	2	42/1964	0.76 [0.21, 2.78]; *P* = 0.68	I^2^ = 83%; *P* = 0.01
Combined with CBD stone	2	58/207	3.67 [2.58, 5.20]; *P* < 0.01	I^2^ = 42%; *P* = 0.19
Intraoperative biliary injury	3	4/13	2.01 [0.63, 6.41]; *p* = 0.24	I^2^ = 38%; *P* = 0.20
Postoperative complication				
Bile leakage	2	5/9	19.00 [5.44, 66.41]; *P* < 0.01	I^2^ = 0%; *P* = 0.78
Wound infection	3	5/10	11.23 [3.75, 33.67]; *P* < 0.01	I^2^ = 0%; *P* = 0.99
Bleeding	3	1/10	4.28 [0.73, 25.32]; *p* = 0.11	I^2^ = 0%; *P* = 0.80

*Abbreviations*: *95% CI* 95% confidence intervals, *CBD* common bile duct

### Surgical details and outcomes

**Fig. 2:**
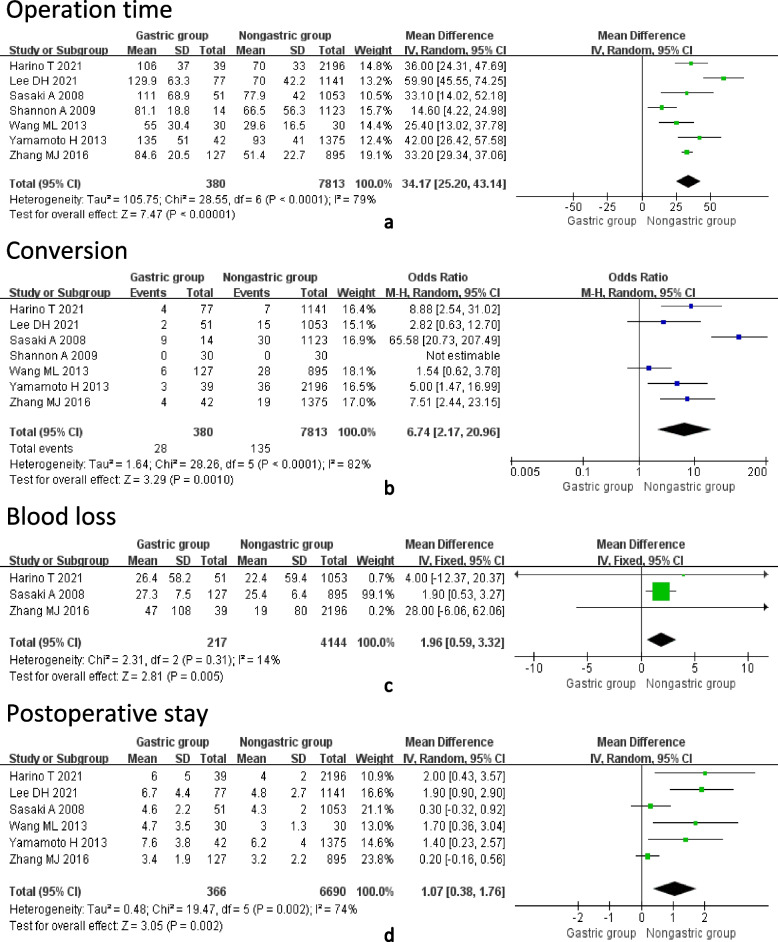
Forest plot showing the surgical-related information. **a** Operation time, **b** Conversion, **c** Blood loss, **d** Postoperative stay

Operation time, conversion rate, intraoperative blood loss, and postoperative hospital stays were compared between the different groups. After pooling up analysis, we found that the gastrectomy group had longer LC operation time (MD = 34.17, 95%CI = 25.20–43.14, *P* < 0.01), a higher conversion rate (OR = 6.74, 95%CI = 2.17–20.26, *P* = 0.01), more intraoperative blood loss (OR = 1.96, 95%CI = 0.59–3.32, *P* < 0.01), and longer postoperative hospital stays (MD = 1.07, 95%CI = 0.38–1.76, *P* < 0.01) than the non-gastrectomy group (Fig. 2).

### Sensitivity and publication bias

**Fig. 3:**
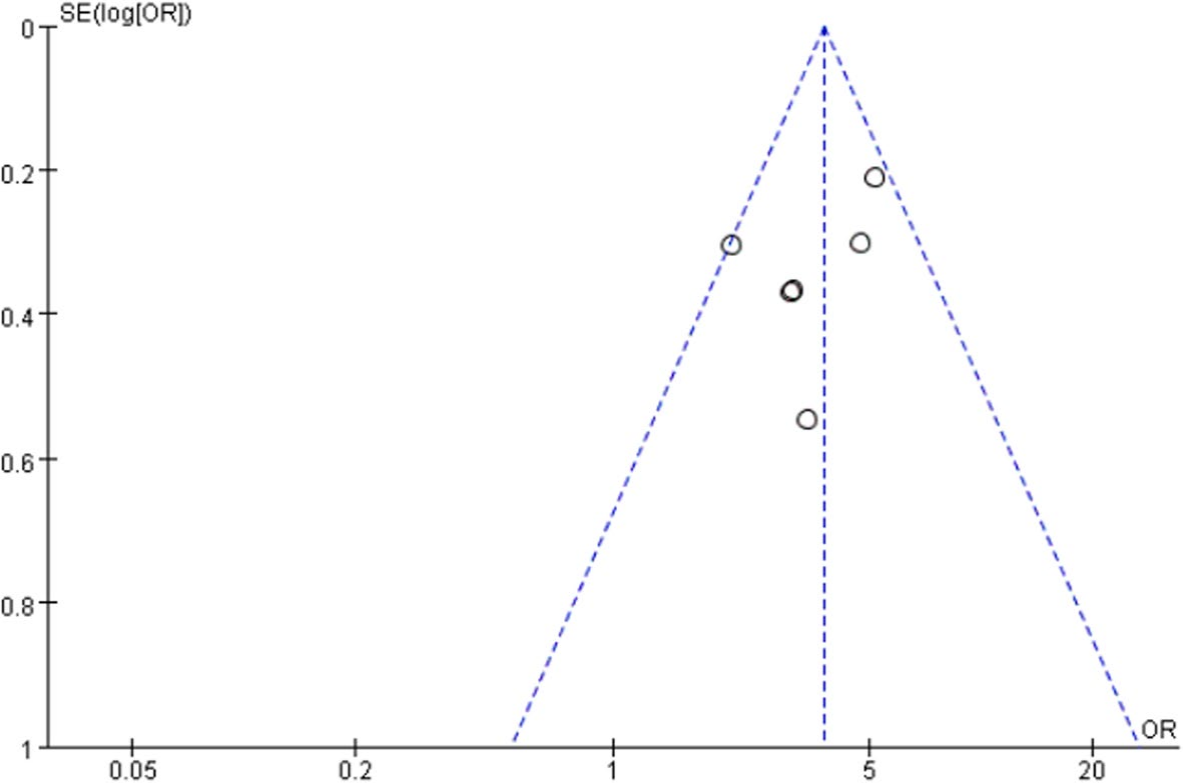
Funnel plot of the included studies

Repeated meta-analysis was performed by excluding each study at a time, and no significant difference was found in each outcome. To evaluate the publication bias, the funnel plot was conducted, and no obvious bias was found (Fig. 3).

## Discussion

This study identified seven studies including 8193 patients. In terms of baseline information, the gastrectomy group were older and had a higher portion of males than the non-gastrectomy group. As for the surgical outcomes, we found that the gastrectomy group had longer operation time, a higher conversion rate, more intraoperative blood loss, and longer postoperative hospital stays.

Comparing the traditional OC, LC had a milder incision, earlier recovery of bowel function, and shorter postoperative hospital stays [25, 26]. However, previous upper abdominal surgery might affect the surgical procedure for the second LC [27, 28], which was considered as a relative contraindication in the past [29]. Gastrectomy was an indication for some gastric diseases, including gastric cancer, gastric ulcer, gastric perforation et al. [30–32]. Some research demonstrated that previous upper abdominal surgery correlated with poor surgical outcomes while undergoing LC [29, 33], but there existed a controversy for the previous gastrectomy.

A retrospective study enrolled 2235 patients and revealed that patients with a previous gastrectomy history were associated with a higher conversion rate while undergoing LC surgery [18]. Some other research also found similar result [16, 17, 19]. However, Sasaki et al. [21] reported that there was no significant difference between the patients with and without previous gastrectomy in conversion rate. The same conclusion was also found by Zhang et al. [20]. As for the postoperative hospital stays, Sasaki et al. [19] and Zhang et al. [20] claimed that patients with a previous gastrectomy history didn’t prolong their postoperative hospital stays, which was opposite of other research [16–18, 24]. One of the most usual reasons for converting from LC to OC was that the previous gastrectomy history might lead to adhesions [34, 35]. If there were some important organs adhering to the LC surgical area, inserting trocars might injury the important organs, which led to a high conversion rate [36, 37]. Therefore, a thorough preoperative examination is necessary, especially for patients with previous gastrectomy [38–40].

To our knowledge, this pooling up analysis was the first one to compare the surgical outcomes between patients with and without a previous gastrectomy. However, some limitations still existed in our study. First, six of the included studies were from Asia, and one was from North America, which might lead to a region restriction. Second, the sample size was relatively small. Third, the information on patients combining with CBD stones was incomplete. Therefore, more detailed studies were needed for further exploration.

In conclusion, patients with a previous gastrectomy history had longer operation time, a higher conversion rate, more intraoperative blood loss, and longer postoperative hospital stays than patients without it while accepting LC. A thorough preoperative examination and comprehensive evaluation were necessary.

## Data Availability

The datasets used and analyzed during the current study are available from the corresponding author on reasonable request.
